# Inhibition of food craving is a metabolically active process in the brain in obese men

**DOI:** 10.1038/s41366-019-0484-z

**Published:** 2019-11-18

**Authors:** Gene-Jack Wang, Ehsan Shokri Kojori, Kai Yuan, Corinde E. Wiers, Peter Manza, Christopher T. Wong, Joanna S. Fowler, Nora D. Volkow

**Affiliations:** 1 Laboratory of Neuroimaging, National Institute on Alcohol Abuse and Alcoholism, Bethesda, MD 20892-1013 USA; 2 School of Life Science and Technology, Xidian University, Xi’an, 710071 Shaanxi China; 3 Brookhaven National Laboratory, Upton, NY 11973 USA; 4 National Institute on Drug Abuse, National Institutes of Health, Bethesda, MD 20892 USA

**Keywords:** Cognitive control, Translational research

## Abstract

**Objective:**

Obesity is associated with impaired inhibitory control over food intake. We hypothesized that the neural circuitry underlying inhibition of food craving would be impaired in obesity. Here we assessed whether obese men show altered brain responses during attempted cognitive inhibition of craving when exposed to food cues.

**Methods:**

Sixteen obese men (32 ± 8.7 years old, BMI = 38.6 ± 7.2) were compared with 11 age-matched non-obese men (BMI 24.2 ± 2.5) using PET and FDG. Brain glucose metabolism was evaluated in a food deprived state: no food stimulation, food stimulation with no inhibition (NI), and food stimulation with attempted inhibition (AI), each on a separate day. Individualized favorite food items were presented prior to and after FDG injection for 40 min. For AI, participants were asked to attempt to inhibit their desire for the food presented. Self-reports for hunger and food desire were recorded.

**Results:**

Food stimulation compared with no stimulation increased glucose metabolism in inferior and superior frontal gyrus, default mode network and cerebellum, in both groups. For both groups, AI compared with NI-suppressed metabolism in right subgenual anterior cingulate, orbitofrontal areas, bilateral insula, and temporal gyri. There was a stimulation-by-group interaction effect in obese (but not in non-obese) men showing increased metabolism in pregenual anterior cingulate cortex (pgACC) and caudate during AI relative to NI. Changes in the food desire from NI to AI correlated negatively with changes in metabolism in pgACC/caudate in obese but not in non-obese men.

**Conclusions:**

Obese men showed higher activation in pgACC/caudate, which are regions involved with self-regulation and emotion/reward during AI. Behavioral associations suggest that successful AI is an active process requiring more energy in obese but not in non-obese men. The additional required effort to increase cognitive control in response to food stimulation in obese compared with non-obese men may contribute to their uncontrolled eating behavior.

## Introduction

Obesity is associated with significant increases in morbidity and mortality, highlighting the urgency for understanding the processes contributing to this epidemic [1, 2]. In many of the current environments, highly palatable and affordable food is widely available. The capacity for self-regulation is likely to modulate an individual’s risk for overeating. Indeed the capacity to regulate impulses, emotions, and desires has been linked to a broad spectrum of psychopathologies and problems including obesity [3–5].

We previously showed that the desire for food during presentation of palatable food stimuli was associated with striatal dopamine (DA) release, measured using positron emission tomography (PET) with [^11^C]raclopride, a radiotracer whose binding is sensitive to changes in extracellular DA. This finding is consistent with DA’s role in modulating the motivation for food [6], which is mediated by its regulation of striato-cortical circuits involved with drive, salience attribution, and inhibitory control [7, 8]. Using the same experimental paradigm in normal-body-weight fasting subjects with 2-deoxy-2[^18^F]fluoro-D-glucose (FDG)-PET, we found that food presentation increased metabolism in orbitofrontal cortex (OFC) in proportion to the subjective perception of hunger and the desire to eat [9]. The OFC is implicated in controlling and planning behaviors and is regulated by DA both through direct as well as indirect striato-thalamo cortical projections. Indeed, a recent fMRI study using the blood oxygen level dependent signal showed that obese subjects activated striatum and OFC as well as insula (a brain region involved with interoception for food signals that is also innervated by DA terminals) while viewing pictures of high-caloric food [10].

Food is a potent natural reinforcer, the value of which is enhanced by food deprivation [11]. Understanding the neurobiological mechanisms underlying the inhibitory control of food intake when one desires it, may provide new targets for interventions to help individuals regulate their eating behaviors and maintain a healthy BMI. We previously reported on a PET study in normal weight participants in whom we measured regional brain glucose metabolic responses when exposed to appealing food both when directed to suppress the food desire (attempted inhibition or AI) and when exposed to the food but with no inhibition (NI) [12]. We showed that AI decreased the activation responses in anterior cingulate cortex (ACC), OFC, striatum and insula to food stimulation in men but not in women [12]. However, the implications of these findings to obesity remains elusive, particularly since healthy obese subjects have lower baseline (BL) metabolic activity in ACC and OFC that was associated with impaired performance on tests of cognitive control [13]. In this study, we used the same imaging paradigm as reported previously [12] in a group of obese (*n* = 16) and non-obese men (*n* = 11). We hypothesized, first, obese individuals would have decreased activation in OFC, striatum, and insula but increases in ACC during AI, as compared with controls. Second, we expected differential metabolic responses to AI compared with NI based on group (i.e., a group-by-condition interaction in metabolic activity), based on several studies suggesting obesity-related differences in brain functional activity when regulating responses to food [10] (for a review, see [14]). Finally, in line with substantial individual differences in brain responses and outcomes during self-control for food [15, 16], we expected that metabolic activity changes in AI versus NI would be significantly associated with suppression of hunger and food desire in AI relative to NI.

## Materials and methods

### Participants

The Institutional Review Board at Stony Brook University/Brookhaven National Laboratory approved the protocol. Written informed consents were obtained after the experimental procedure was explained and after the participants had read the consent form. Sixteen obese males (32 ± 8.7 years old) with mean BMI of 38.6 ± 7.2 and 11 age-matched non-obese males (BMI 24.2 ± 2.5) were recruited for the study. Obese participants had a BMI of 30 or over: 12 of them with BMI range (31.1–38.9), four of them were considered morbidly obese (43.1–60). Whereas the BMI of non-obese was below 30, six non-obese participants were considered normal weight (20.2–24.5) and five non-obese participants were considered overweight (25.1–29.6). Participants with the following conditions were excluded: past or present history of eating disorders as per DSM IV, surgical/medical treatment for weight control, dependence on alcohol or other drugs of abuse (except for caffeine < 5 cups/day or nicotine < 1 pack/day), neurological or psychiatric disorder, use of prescription (psychiatric and/or non-psychiatric) medication(s) that can affect brain function in the past 2 weeks, medical conditions that may alter cerebral function, cardiovascular disease and diabetes, and head trauma with loss of consciousness of more than 30 min. Urine screening tests for psychoactive drugs (including PCP, cocaine, amphetamine, opiates, barbiturates, benzodiazepine, and THC) were performed to corroborate absence of drug use. Sample size was justified based on a prior study with a similar number of participants in which there was sufficient statistical power for detecting food inhibition effects [12].

Prior to the imaging studies, participants were asked to fill out a questionnaire, which contained the following information: a rating of their interest in food; their favorite foods; food smells that stimulated their appetite; food smells that diminished their appetite; and a list of foods to rate for their preference on a scale from 1 to 10, 10 being the highest. The food items included a variety of popular American meals, snacks and desserts (e.g., bacon–egg–cheese sandwich, cinnamon bun, pizza, hamburger with cheese, fried chicken, lasagna, barbecue ribs, ice cream, brownie, and chocolate cake). The ten food items with the highest ratings were presented to the participant on the imaging study day prior to the food stimulation to confirm their favorite choices and during the food stimulation condition. To assess the behavioral dimensions of eating, participants also completed the Three Factor Eating Questionnaire (TFEQ) [17], which has three main factors (disinhibition, cognitive restraint of eating, and susceptibility to food cues). Since the participants had to fast for 15 or more hours before the PET imaging was done, these questionnaires were filled on a screening day under ‘normal’ non-stressful circumstances.

### Experimental paradigm

Participants were scanned three times with FDG in three different days under the following conditions: (1) Day A: food stimulation started 15 min before FDG injection and continued for a total of 45 min while lying in the PET scanner. When the stimulation was completed, the participants were positioned for imaging and acquisition was started 35 min after FDG injection. The participants were instructed to observe and spontaneously react to the food stimuli (no inhibition: NI). (2) Day B: procedures were as for Day A except that participants were instructed to inhibit their food desire prior to the presentation of the food stimuli (attempted inhibition: AI). Prior to the study, participants were instructed how to practice their attempted inhibition (i.e., ignore, shift thoughts) to the food stimuli. (3) Day C: BL condition without food stimulation. The A, B, and C sequence was randomized so that for one third of the participants the first day was day A, for one third it was day B and for the other third it was day C.

For day A and day B, the food was warmed to enhance the smell and the participants were presented with it so that they could view it and smell it by one of the investigators (M.J.). A cotton swab impregnated with the food was placed on their tongue so they could taste (but not swallow) it. A given food item was presented for 5 min and then it was exchanged for a new one. The tasting, smelling, and viewing of a given food item were continuous during the stimulation. The participants were asked to describe their favorite foods and how they like to eat them while they were presented with foods that they had reported as among their favorite ones. For all conditions, participants were asked to have their last meal at 7 P.M. the evening before the day of the study and were studied between 17 and 19 h after their last meal. We relied on the participants’ self-report to confirm that they had not eaten anything after their last meal as instructed. The participants arrived at the imaging center at about 8:30 A.M. on the day of the study and a nurse remained with them to ensure they refrained from food or caloric containing drinks prior to imaging, which started after 12 P.M.

### PET imaging

Participants were scanned with FDG using a Siemens HR + PET scanner. Details on procedures for positioning of the participant, arterialized venous and venous catheterization, quantification of radiotracer and transmission and emission scans have been published [9]. Briefly, two intravenous lines were inserted and maintained with saline. Arterialized venous blood was obtained from a catheter placed in a dorsal hand vein. The hand was prewarmed to 48 °C in a heating box, which ensured the shifting of arterial blood to the vein. The other catheter was in the antecubital region of the opposite arm for tracer injection. One emission scan (20 min) was taken 35 min after an intravenous injection of 4–6 mCi of FDG. During the study participants were positioned supine in the PET camera with their eyes open; the room was dimly lit and noise was kept to a minimum.

### Behavioral assessment

During the PET studies participants were instructed to orally respond to each descriptor using a whole number between 1 and 10 for the self-report of hunger and food desire, which were obtained during food presentation in 5 min intervals for a total of 45 min (ten samples). The measures from 25 to 45 min where averaged (Supplementary Fig. 1).

### Data analysis

PET images were reconstructed using filtered back projection (Hann filter with a 4.9 mm FWHM kernel). Cerebral glucose metabolic rate (CMRglu) images were computed using Sokoloff’s model [18]. CMRglu images were transferred into MNI space in SPM8 (Wellcome Trust Centre for Neuroimaging, London) [19]. SPM8 was used to perform voxelwise analysis of variance (ANOVA) using the full factorial model to study the within-subject factor conditions (with BL, NI, and AI levels) and the between-subject factor of groups (obese and non-obese) and their interactions. An uncorrected threshold of *p* = 0.002 and minimum cluster size of 200 was applied to the statistical maps in SPM8. When indicated, correction for multiple comparisons was performed using random field theory for cluster size relative to whole brain or a small volume.

Behavioral ratings of hunger and food desire were analyzed using a repeated measures ANOVA with condition (NI and AI) as within-group factor, and group (obese and non-obese) as between-group factor, using SPSS version 25 (IBM). Pearson product moment correlation analyses were used to assess the association between the ratings of hunger and food desire and the condition-related changes in CMRglu.

## Results

Table 1 provides demographics of the obese and non-obese group. Obese participants did not differ from the non-obese in age or years of education. The obese participants had higher weights and BMI, and scores higher on the TFEQ score (total), driven by their higher subscores in disinhibition and cognitive restraint of eating (see Table 1).

**Table 1:** Characteristics of study participants

	Obese	Non-obese	*p* value
Number of participants	16 Men	11 Men	
Age range (years old)	21–46	24–45	
Age mean (years old)	31.9 ± 8.7	31.2 ± 5.8	NS
Body weight range (lb)	192–480	137–200	
Body weight mean (lb)	269 ± 67	166 ± 20	<0.001
BMI range (kg/m^2^)	31.1–60	20.2–29.6	
BMI mean (kg/m^2^)	38.6 ± 7.2	24.4 ± 2.6	<0.001
Education (years)	13.5 ± 2.0	14.2 ± 2.2	NS
TFEQ total	7.2 ± 1.8	5.6 ± 1.8	0.001
TFEQ cognitive restraint of eating	8.2 ± 4.3	4.4 ± 3.3	0.03
TFEQ disinhibition	8.4 ± 3.5	3.7 ± 2.1	<0.001
TFEQ hunger	8.9 ± 3.2	6.7 ± 3.5	0.1

### Behavioral ratings

The repeated measures ANOVA showed a significant effect of condition (NI and AI) on rating of hunger ratings (*F*_1,25_ = 56.0, *p* < 0.0001) and food desire (*F*_1,25_ = 50.0, *p* < 0.0001). During AI, both non-obese and obese men had lower hunger (*t*_26_ = 7.3, *p* < 0.0001) and food desire (*t*_26_ = 7.1, *p* < 0.0001) scores compared with NI (also see Supplementary Fig. 1). There was no effect of group and no interaction effect of group × condition on ratings of hunger or food desire (Table 2).

**Table 2:** Comparison between obese and non-obese men for the difference in the scores (from 1 to 10; 0 is the least and 10 is the most) of self-report of hunger and food desire during food stimulation conditions

	Hunger			Food desire		
	Obese	Non-OB	*p* value	Obese	Non-OB	*p* value
No inhibition (NI)	8.6 ± 1.0	8.8 ± 0.8	NS	8.3 ± 1.1	8.4 ± 1.1	NS
Attempted inhibition (AI)	5.5 ± 2.6	4.5 ± 2.9	NS	5.1 ± 2.5	4.4 ± 2.9	NS
AI–NI	−3.2 ± 2.3	−4.3 ± 2.9	NS	−3.2 ± 2.6	−4.0 ± 2.6	NS
NI vs AI (*p* value)	<0.001	<0.001		<0.001	<0.001	

*NI* food stimulation without attempted inhibition, *AI* food stimulation with attempted inhibition

### Metabolic changes between baseline and non-inhibited food stimulation

**Fig. 1:**
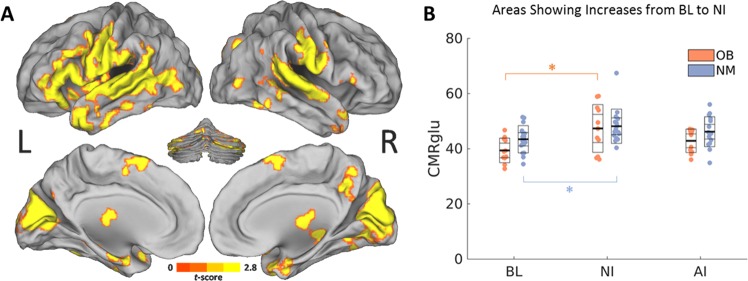
Effect of food stimulation. **a** Brain regions showing CMRglu increases in food stimulation with no inhibition (NI) relative to baseline (BL), *p* = 0.002 uncorrected, minimum cluster size = 200. Also see Table 1. **b** Group average of CMRglu within areas shown in **a** in BL, no inhibition (NI), and attempted inhibition (AI) conditions. Significant differences are shown with brackets

In the no-stimulation BL, CMRglu was lower in a cluster (*k* = 246) within the left medial and superior frontal gyri (BA 9, peak coordinates: *x* = −24 mm, *y* = 18 mm, and *z* = 36 mm) in obese than non-obese men (Supplementary Fig. 2). Relative to BL, the food stimulation with no inhibition (NI), had increases in CMRglu in a range of cerebellar, cortical and subcortical regions (Table 3, Fig. 1a). These increases were significant in both obese and non-obese groups (Fig. 1b). However, there were no significant differences in food stimulation with NI induced metabolic increases between obese and non-obese groups.

**Table 3:** Food stimulation (without inhibition) versus baseline condition (both groups)

Region(s)	L/R	Brodmann area(s)	Cluster size	Peak coordinates (*x*, *y*, *z*)_mm_			Peak *t*-value
Cerebellum ant lobe Cerebellum post lobe Declive Cuneus Middle temporal gyrus Sup. temporal gyrus Calcarine Tuber Cerebellar tonsil Precentral gyrus Postcentral gyrus Precuneus Culmen	L/R	18, 22	*15345	−30	−58	−30	6.10
Sup. temporal gyrus Middle temporal gyrus Inf. temporal gyrus	L	21, 38, 20	*724	−38	14	−32	4.18
Middle temporal gyrus Sup. temporal gyrus Uncus Inf. frontal gyrus Parahippocampus	R	38, 47	*365	28	2	−34	4.10
Parahippocampus Uncus	L	34, 28	206	−18	−14	−24	4.01
Inf. frontal gyrus Sup. temporal gyrus	R	47, 38	261	54	24	−2	4.05
Sup. temporal gyrus Inf. frontal gyrus Middle temporal gyrus Precentral gyrus Postcentral gyrus Insula Middle frontal gyrus Inf. parietal lobule	L	22, 40, 21	*10042	−56	−12	24	7.01
Thalamus Medial dorsal nucleus Midbrain	L/R	–	*771	6	−18	4	4.35
Precuneus	R	7, 31	213	10	−58	30	4.17
Superior frontal gyrus	L/R	6	*391	−8	12	60	5.07

*p* = 0.002, uncorrected, minimum cluster size = 200

*p_FWE_ < 0.05 cluster-size corrected, random field theory

### Metabolic changes between non-inhibited food stimulation and attempted inhibition

**Fig. 2:**
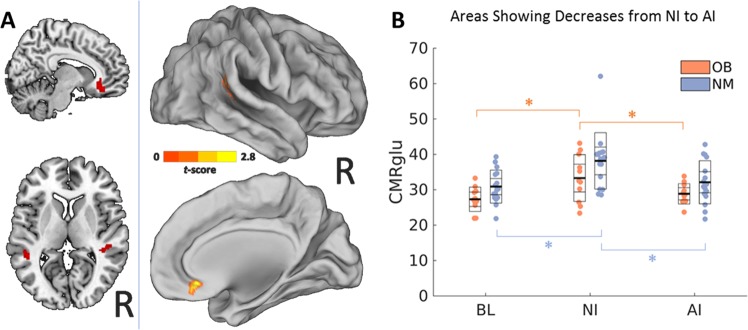
Effect of attempted inhibition (AI) in non-obese (NM) and obese (OB) individuals. **a** Brain regions showing decreases in CMRglu in active inhibition (AI) relative to no inhibition (NI) in both groups (*p* = 0.002 uncorrected, minimum cluster size = 200). Also see Table 2. **b** Group average of CMRglu within areas shown in **a** in BL, no inhibition (NI), and attempted inhibition (AI) conditions

Relative to NI, AI in both non-obese and obese groups decreased CMRglu in middle and superior temporal gyrus, right insula, right subgenual anterior cingulate (sgACC) and right medial orbitofrontal cortex (mOFC, Table 4, Fig. 2a), while the same regions had higher CMRglu in NI relative to BL (Fig. 2b).

**Table 4:** Attempted inhibition versus without inhibition to food stimulation condition (both groups)

Region(s)	L/R	Brodmann area(s)	Cluster size	Peak coordinates (*x*, *y*, *z*)_mm_			Peak *t*-value	
Middle temporal gyrus Sup. temporal gyrus	L	22, 21	*381	−48	−38	2		4.58
Medial frontal gyrus Subgenual Ant. cingulate (sgACC) Orbitofrontal gyrus	R	32, 10	215	20	34	−4		4.02
Sup. Temporal Gyrus Insula	R	41, 13	215	56	−38	18		4.11

*p* = 0.002, uncorrected, minimum cluster size = 200

*p_FWE_ < 0.05 cluster-size corrected, random field theory

### Interaction between brain glucose metabolism and attempted inhibition

**Fig. 3:**
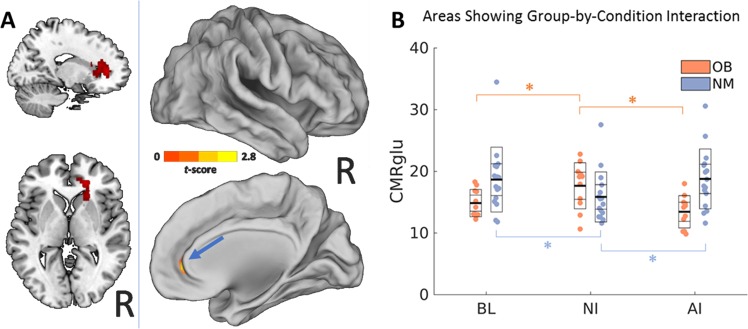
Interaction between inhibition to food stimulation and group factors in CMRglu. **a** Brain regions showing different changes from food stimulation with no inhibition (NI) to food stimulation with attempted inhibition (AI) in normal controls (NM) versus obese (OB) participants (*p* = 0.002 uncorrected, minimum cluster size = 200). Also see Table 3. **b** Group average of CMRglu within areas shown in **a** in BL, no inhibition (NI), and attempted inhibition (AI) conditions

For the interaction between-group and experimental condition, we found a cluster (BA32) within the pgACC and caudate with differential involvement in NI versus AI for the obese and non-obese groups (Table 5, Fig. 3). In the non-obese group, we found significant increases from BL to NI and significant decreases from NI to AI in this cluster (Fig. 3b). In the obese group, however, we found significant decreases from BL to NI and increases from NI to AI in this cluster (Fig. 3b). There was no significant group interaction effect for CMRglu changes between BL and NI.

**Table 5:** Interaction between attempted inhibition and without inhibition to food stimulation between two groups

Region(s)	L/R	Brodmann area(s)	Cluster size	Peak coordinates (*x*, *y*, *z*)_mm_			Peak *t*-value	
Pregenual Ant. Cingulate (pgACC) Caudate	R	32	^a^278	20	38	4		5.18

*p* = 0.002, uncorrected, minimum cluster size = 200

^a^Correction for multiple comparisons using small volume correction in a sphere with a 50 mm radius within the frontal cortex, *p*_FWE_ < 0.05

Interestingly, the CMRglu during AI in the left dorsolateral prefrontal cortex (DLPFC) cluster (with significant BL group differences), showed a trend correlation with the pgACC cluster across non-obese (*r*(9) = 0.59, *p* = 0.056) but not across obese men (*r*(14) = 0.36, *p* = 0.17).

### Correlation between brain glucose metabolism and behavioral ratings

**Fig. 4:**
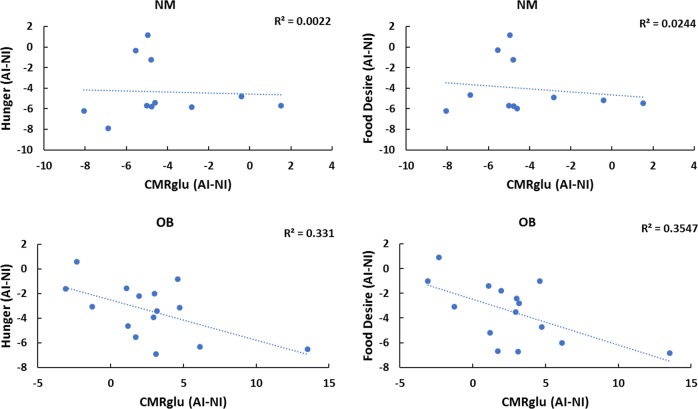
Correlation between changes in the behavioral measures and CMRglu in the cluster showing interaction effect between-group and inhibition to food stimulation factors (Fig. 3). One outlier participant for behavioral assessment was excluded (see Supplementary Fig. 2). NI food stimulation without inhibition, AI food stimulation with attempted inhibition, OB obese, and NM non-obese controls. Both correlations were significant for the OB group. OB participants with more hunger suppression increased CMRglu

In the obese group, condition-related differences in ratings of hunger and food desire were associated with condition-related changes in CMRglu. Specifically, there was a negative association between changes in ratings from NI to AI and CMRglu changes in pgACC/caudate (Hunger: *R*^*2*^ = 0.33, *p* = 0.020; Food desire: *R*^*2*^ = 0.35, *p* = 0.015; Fig. 4; also see Supplementary Fig. 3); such that greater suppression of appetite was associated with higher pgACC/caudate activation (cluster showing the interaction effect, Fig. 3; Table 5). In the non-obese participants, this association was not significant (Hunger: *R*^*2*^ = 0.002, *p* = 0.896; Food desire: *R*^*2*^ = 0.02, *p* = 0.650; Fig. 4).

**Fig. 5:**
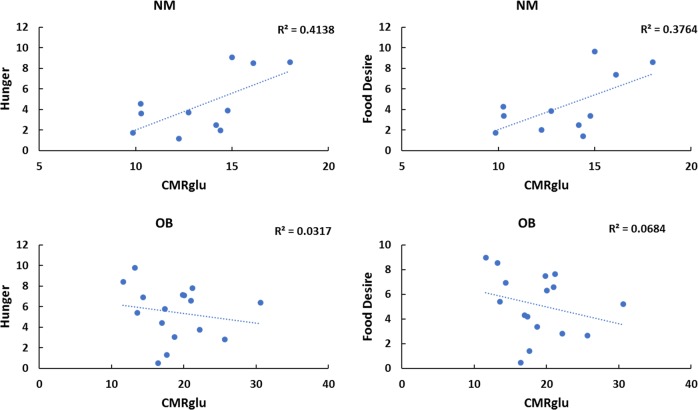
Correlations between behavioral measures and CMRglu during AI in the cluster showing interaction effect between-group and inhibition to food stimulation factors (Fig. 3). NI food stimulation without inhibition, AI food stimulation with attempted inhibition, OB obese, and NM non-obese controls. Both correlations were significant only for the NM group

In non-obese participants, for both hunger (*R*^*2*^ = 0.41, *p* = 0.034) and food desire (*R*^*2*^ = 0.38, *p* = 0.045), lower ratings during AI were associated with lower pgACC/caudate metabolism (Fig. 5) for the cluster showing the interaction effect in Fig. 3. This is consistent with non-obese participants showing less activation in this cluster for AI than NI (Fig. 3, Table 5).

**Fig. 6:**
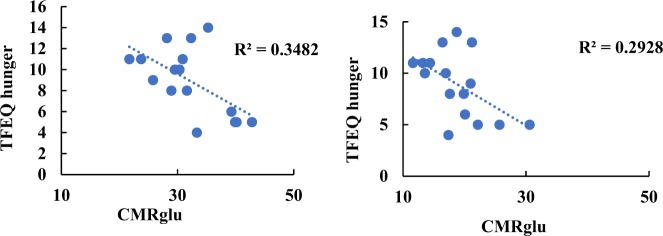
Association between TFEQ-hunger measures and CMRglu changes in food inhibition network (including sgACC, OFC, insula, and temporal cortex) during AI condition (Fig. 6a) and with CMRglu changes for interaction effect (including pgACC and caudate) between groups and inhibition condition (Fig. 6b) in obese participants

We did not observe a correlation between changes in CMRglu and the TFEQ-disinhibition and TFEQ-cognitive restraint of eating measures neither in the obese nor in the non-obese groups. However, the correlation between the TFEQ-hunger measures and CMRglu during AI in the regions activated during AI > NI (sgACC, mOFC, posterior insula/superior temporal gyrus, and middle temporal gyrus) revealed a negative association in obese but not in non-obese participants (*R*^*2*^ = 0.35, *p* = 0.016; Fig. 6a). The TFEQ-hunger scores were also negatively associated with CMRglu in the cluster showing an interaction effect during AI (pgACC and caudate; *R*^*2*^ = 0.29, *p* = 0.030; Fig. 6b), in obese but not in non-obese participants.

## Discussion

This study explored the regional brain metabolic activity, which is presumed to reflect brain activity during food stimulation with and without attempted inhibition in obese men compared their response with those in non-obese men. The findings show that while brain responses to food stimulation with NI were similar between obese and non-obese men, regional brain responses to AI of food craving differed between the obese and the non-obese group. We discuss these findings in more detail below.

### Effects of food stimulation without inhibiting food craving

The SPM comparison between BL and food stimulation (NI) conditions showed that both groups significantly increased CMRglu in what we describe as a “food stimulation network” that included inferior and middle frontal and temporal cortices, precuneus, insula, thalamus, and cerebellum. This finding replicates our previous work [9], and suggests that there may not be significant differences between non-obese and obese men in the regional brain’s metabolic responses to the combination of viewing, smelling and tasting food generally. Participants in both groups fasted for 15 or more hours before the PET FDG scan, which may have minimized group differences in hunger drive. Thus, the positive finding in the current study is supportive of an actual effect of obesity on brain glucose metabolism responses.

### Effects of food stimulation during attempted inhibition of food craving

Similar to our prior findings in non-obese males [12], obese participants showed lower CMRglu in sgACC/mOFC (BA 32, 10), and bilateral posterior insula/superior temporal and middle temporal gyri during AI compared with NI. These regions are notable for their involvement in multisensory integration, as well as reward and emotional processing [20–22]. The sgACC is of particular interest, since it serves as a functional nexus for reward, emotion, and cognitive processes [23]. For instance, when a cognitive control task was carried out in an emotional context or when the emotional stimuli were relevant to the cognitive task being carried out, the sgACC was activated [24]. The ventral medial PFC is also involved in self-control and decision making processes as part of a circuit including DLPFC. The sgACC encodes stimulus values that guide decisions at the time of choice, relaying these values to dLPFC [25]. The DLPFC could filter sensory input then modulate and incorporate these value signals for successful cognitive control during decision making, making this a likely circuit that regulates food intake behavior.

Empirical studies have shown support for this possibility. For example, decision task studies suggest that the DLPFC influences self-control by modulating sgACC value signals to reflect more desirable elements such as the health value, rather than more attractive but inferior rewards such as taste value of foods [26–28]. Based on this work, our observed suppression of sgACC CMRglu during AI in the non-obese group may reflect suppressed hunger and food desire. Notably, in this study, the food stimulation was not passive viewing of food cues but actively viewing, smelling and tasting preferred food items simultaneously that were individually selected by each participant prior to study day. This multisensory stimulation procedure recruited brain regions involved with sensory processing, emotional regulation, conditioning and motivation, and these same regions were suppressed during AI.

### Interactive effects of obesity and inhibition of food craving on brain glucose metabolism

For the interaction between-group and inhibition conditions, we found a cluster including parts of the pgACC and caudate that had differential involvement in NI versus AI for obese and non-obese groups. Specifically, in non-obese participants, this cluster was significantly increased from BL to NI and decreased from NI to AI, whereas OB participants increased CMRglu in this region from NI to AI. The pgACC area is known to be activated by many reward related stimuli including fat texture and sweet taste [22, 29]. This region involves action outcome learning process and is activated when an expected reward delays [30]. The caudate is associated with reward anticipation [31, 32] and initiation of eating behavior [33], which has been shown to be activated in patients with binge eating disorder during food stimulation condition using the same food stimulation paradigm of this study [34]. The increases of CMRglu in pgACC/caudate cluster of obese participants could reflect greater energy need in this region during the inhibition control task. In obese men (but not non-obese), changes in the rating of hungry and food desire ratings from NI to AI was negatively correlated with CMRglu changes in the pgACC/caudate cluster showing an interaction effect. Thus, successful AI in obese participants was related to recruitment of additional metabolic activation in this cluster. Decreased CMRglu in frontal cortex has been shown in obese participants, which is associated with decreased cognitive function [13]. The decreases of CMRglu in PFC (including ACC) in morbidly obese participants was associated with deficits in DA D2 receptor mediated striatal signaling (including caudate) [8]. In this study, we also observed that the obese participants had lower BL CMRglu in left DLPFC. We speculate that obese participants have greater difficulty in suppressing their desire to eat during food stimulation, for it is a more demanding cognitive effort that appears to require a greater energetic cost for activating the pgACC/caudate region which is needed for self-regulation [35].

### Behavioral associations with CMRglu changes during food stimulation and inhibition

In our study, the obese group had higher TFEQ-disinhibition and TFEQ-restraint scores than the non-obese group, but these measures did not correlate with CMRglu. On the other hand, though TFEQ-hunger scores did not differ between the groups, in obese but not in non-obese participants, they were negatively associated with CMRglu during AI in the “food inhibition network” (regions that differed between AI and NI). Activation of sgACC was reported in an fMRI study that compared participants with different TFEQ-disinhibition scores when assessing valuation (greater taste-unhealthy vs. neutral taste-healthy) of food [36]. In this study, participants with lower disinhibition scores attenuated sgACC activity when fed whereas participants with higher disinhibition scores showed enhanced activity. In our study obese men had higher TFEQ-disinhibition scores, which is consistent with prior reports and expected for these scales, which were designed to measure long-term attitudes to eating [37]. The measures of TFEQ-hunger have been associated with a measure of susceptibility to hunger cues. It is likely that obese participants are more susceptible to craving when exposed to food cues under fasting condition, and this craving may be reflected in the regional patterns of brain glucose metabolism observed here.

#### Limitations

Because our prior study demonstrated that female participants did not suppress CMRglu during AI using the same food stimulation paradigm [12], we recruited male participants only in the present study. Thus, the findings in this study may not generalize to female obese participants. Future studies using different food stimulation paradigms are warranted to understand gender differences on attempted inhibition and its effect on food cue induced brain activation. Moreover, we investigated presession food intake using self-reports on the TFEQ. However, a newer version is available that has better validity and facture structure than the TFEQ used in the current study [38]. Furthermore, study data collection took part between 2011 and 2012. As a result, principles and practices of open science including preregistration of hypotheses and planned analyses were not adhered to. In addition, Future studies should attempt to replicate our findings in a larger and more diverse sample of participants. Last, the non-obese group consisted of participants in both the normal weight (BMI 20–25) and overweight (25–30) category.

### Summary

Here we show the suppression of CMRglu in sgACC/OFC, posterior insula and superior and middle temporal gyri during food presentation with AI of food craving in non-obese men. These regions are involved in sensory processing, emotional regulation, conditioning, and motivation, suggesting their role in the neurobiological mechanisms underlying cognitive inhibition of the desire for food. Interestingly, the pgACC/caudate regions in obese men showed an increase in CMRglu from NI to AI (a change that was correlated with better suppression scores), while the non-obese men showed an opposite pattern. The pgACC/caudate regions are involved in processing motivation for food consumption. The additional required effort to increase cognitive control in response to food stimulation in obese compared with non-obese men may contribute to their uncontrolled eating behavior.

## Supplementary information

Supplementary figures
