# Supplementary material for: Inhibition of food craving is a metabolically active process in the brain in obese men
Source: Int J Obes (Lond). 2019 Nov 18;44(3):590–600. doi: 10.1038/s41366-019-0484-z (PMC7046524; doi:10.1038/s41366-019-0484-z)

**Supplementary Material**

**Inhibition of food craving is a metabolically active process in obese men**

**Gene-Jack Wang^1^, Ehsan Shokri Kojori^1^, Kai Yuan^1,2^, Corinde E. Wiers^1^, Peter Manza^1^, Christopher T. Wong^1^, Joanna S. Fowler^3^, Nora D Volkow^1,4^.**

**^1^ Laboratory of Neuroimaging, National Institute on Alcohol Abuse and Alcoholism, Bethesda, MD, 20892-1013**

**^2^ School of Life Science and Technology, Xidian University, Xi'an Shaanxi 710071, Peoples R China**

**^3^ Brookhaven National Laboratory, Upton, NY, 11973**

**^4^ National Institute on Drug Abuse, National Institutes of Health, Bethesda, MD, 20892**

**Correspondence:** Gene-Jack Wang MD, National Institute on Alcohol Abuse and Alcoholism, Laboratory of Neuroimaging, National Institutes of Health, 10 Center Drive, Room B2L124, Bethesda, MD, 20892, USA. E-mail: gene-jack.wang@nih.gov, Phone: +1 301-496-5012

**Supplementary Figure 1.** Dynamic changes in the behavioral measures during food stimulation in no inhibition (NI) and attempted inhibition (AI) in normal and obese participants. Self-reported measurements were collected every 5 min.

**
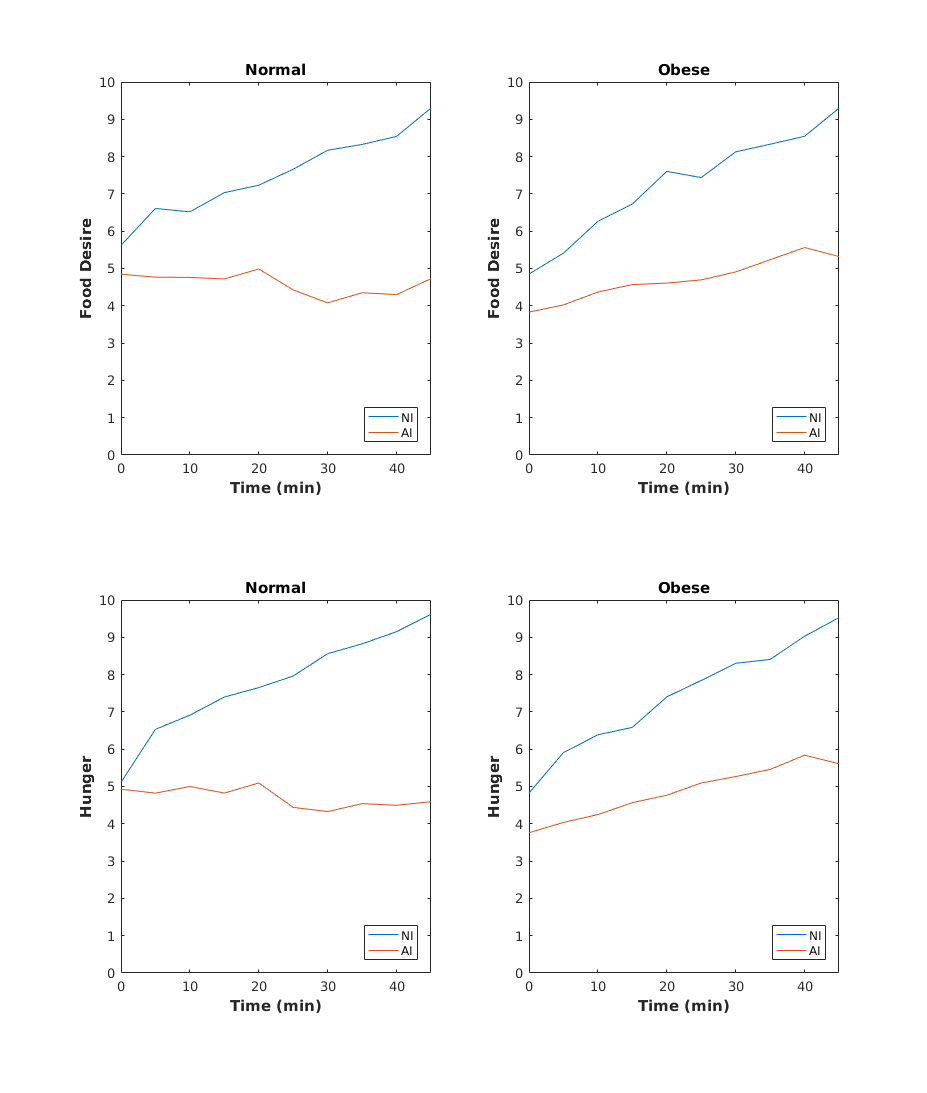
**

**Supplementary Figure 2.** In the no- stimulation baseline (BL), CMRglu was lower within the left medial and superior frontal gyri in obese than non-obese men (*p* < 0.002, k > 200, cluster size = 246).


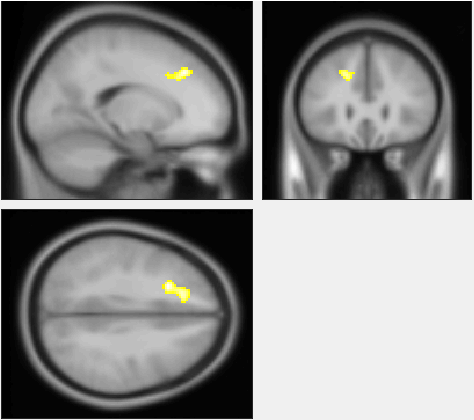


**Supplementary Figure 3.** Outlier detection results for the correlation between changes in CMRglu and changes in subjective ratings of food desire (left) and hunger (right), from no inhibition (NI) to attempted inhibition (AI) conditions.


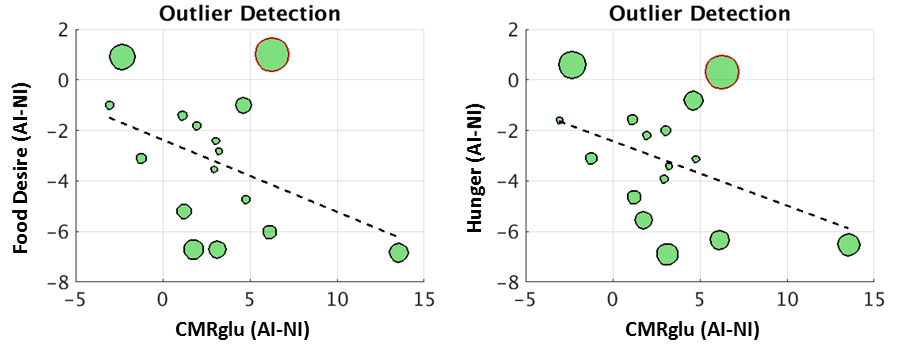

Supplement: Supplementary file 1 — Supplementary figures [file 41366_2019_484_MOESM1_ESM.docx]
